# Predictors of assertive behaviors in Tunisian medical students

**DOI:** 10.1192/j.eurpsy.2025.1832

**Published:** 2025-08-26

**Authors:** M. K. Khamassi, B. N. Saguem, J. Nakhli

**Affiliations:** 1 Department of psychiatry, Farhat hached hospital; 2 Research Laboratory LR12ES04, University of Sousse, Faculty of medicine of Sousse, Sousse, Tunisia

## Abstract

**Introduction:**

Some individuals might be more prone to acquire assertive behaviors earlier in their lifespan than others. Nevertheless, assertiveness can be learnt, and studies suggest that developing them would be beneficial in various fields, including healthcare and medical studies.

**Objectives:**

This study was conducted to assess medical students’ level of assertiveness, and to characterize the factors that might influence it.

**Methods:**

A cross-sectional study was conducted, targeting a representative sample of undergraduate medical students. In total, 91 medical students agreed to participate in an online questionnaire survey. They completed a sociodemographic questionnaire as well as the French validated versions of Rathus Assertiveness Scale (RAS), Depression, Anxiety and Stress Scale, Rosenberg Self-Esteem Scale, General Self-Efficacy.

**Results:**

The response rate was 31.1%. Results showed that 53.8% of participants were considered nonassertive. Assertiveness training was requested by fifty-six students (61.5%). Moderate to extremely severe levels of depression, anxiety and stress were reported by 61.5%, 69.2% and 43.9% of participants respectively. Medical students who practiced individual sport or played music showed significantly lower RAS scores, indicating a higher level of assertiveness (p= .022 and p = .003 respectively). Rosenberg self-esteem scores and general self-efficacy scores were strongly correlated with assertiveness. Depression, anxiety and stress scores were strongly and positively correlated with assertiveness scores. Hierarchical regression analysis revealed that playing music, Self-esteem and self-efficacy were independent predictive variables of assertiveness with beta values of -.219, -.247, and -.209, respectively, explaining 44% of the model.

**Conclusions:**

Assertive communication can provide medical students with an opportunity to become more effective in dealing with those around them, fostering better relationships, and promoting mental well-being. Reinforcing music and individual sports activities may serve as valuable preventive and interventional strategies to improve assertiveness in medical students.

**Disclosure of Interest:**

None Declared

